# Age-Dependent Toxicity Patterns in Medulloblastoma: A Comparative Analysis of Treatment-Related Adverse Events Between Pediatric and Adult Populations

**DOI:** 10.3390/jcm15156023

**Published:** 2026-08-03

**Authors:** Antonio Ruggiero, Dario Talloa, Alberto Romano, Palma Maurizi, Stefano Mastrangelo, Tommaso Verdolotti, Silvia Mariani, Silvia Chiesa, Rina di Bonaventura, Luca Massimi, Gianpiero Tamburrini, Alessio Albanese, Giorgio Attinà

**Affiliations:** 1 Pediatric Oncology Unit, Fondazione Policlinico Universitario Agostino Gemelli IRCCS, 00168 Rome, Italy; dario.talloa@guest.policlinicogemelli.it (D.T.); alberto.romano@policlinicogemelli.it (A.R.); palma.maurizi@unicatt.it (P.M.); stefano.mastrangelo@unicatt.it (S.M.); giorgio.attina@policlinicogemelli.it (G.A.); 2 Department of Women and Child Health and Public Health, Università Cattolica del Sacro Cuore, 00168 Rome, Italy; 3 ARC Advanced Radiology Center (ARC), Department of Oncological Radiotherapy and Hematology, Fondazione Policlinico Universitario Agostino Gemelli IRCCS, 00168 Rome, Italy; tommaso.verdolotti@policlinicogemelli.it; 4 Department of Radiation Oncology, Institute of Radiology, Fondazione Policlinico Universitario Agostino Gemelli IRCCS, Università Cattolica del Sacro Cuore, 00168 Rome, Italy; silvia.mariani@guest.policlinicogemelli.it (S.M.); silvia.chiesa@policlinicogemelli.it (S.C.); 5 Neurosurgery Unit, Department of Neurosciences, Fondazione Policlinico Universitario Agostino Gemelli IRCCS, 00168 Rome, Italy; rina.dibonaventura@policlinicogemelli.it (R.d.B.); alessio.albanese@unicatt.it (A.A.); 6 Pediatric Neurosurgery, Fondazione Policlinico Universitario Agostino Gemelli IRCCS, 00168 Rome, Italy; luca.massimi@policlinicogemelli.it (L.M.); gianpiero.tamburrini@policlinicogemelli.it (G.T.); 7 Department of Neuroscience, Section of Neurosurgery, Università Cattolica del Sacro Cuore, 00168 Rome, Italy; 8 Neurosurgery Unit, Department of Neurosciences, Università Cattolica del Sacro Cuore, 00168 Rome, Italy

**Keywords:** medulloblastoma, chemotherapy toxicity, adult, pediatric, chemotherapy, comparative analysis, relative dose intensity, supportive care

## Abstract

**Background/Objectives**: to compare and characterize the frequency and severity of treatment-related adverse events (TRAEs) experienced by pediatric and adult patients with medulloblastoma receiving the same pediatric-based treatments. **Methods**: a retrospective comparative study of TRAEs was conducted on 18 adult patients and 45 pediatric patients with medulloblastoma treated at the same institution with craniospinal/local radiotherapy and chemotherapy delivered according to pediatric regimens (i.e., PNET3, PNET4, PNET5 or high-risk protocols). **Results**: Grade ≥ 3 hematological TRAEs were significantly more frequent in adults (61.1% vs. 33.3%, *p* = 0.038). Adults also more frequently suffered from weight loss > 10% than children (72.2% vs. 40%, *p* = 0.018). The incidence of chemotherapy-induced peripheral neuropathy did not differ significantly between the two groups (27.7% in adults versus 22.2% in children, *p* = 0.65). These findings suggest that adult patients treated with pediatric-derived protocols may benefit from more proactive hematologic and nutritional supportive care to safely maintain treatment intensity. Objective severe ototoxicity was 22.2% and 17.8% in adults and children (*p* = 0.69), respectively. There were significantly more adults than children who needed a dose reduction (55.6% vs. 28.9% in children, *p* = 0.042) and who stopped treatment prematurely (44.4% vs. 20.1% in children, *p* = 0.048). There was no difference in 5-year overall survival between adults and the pediatric population (88.8% vs. 86.7%, *p* = 0.82). **Conclusions**: Adults are at an increased risk of hematological and nutritional toxicities when treated for medulloblastoma with identical treatment regimens. These age-related patterns of sensitivity demand protocol modifications (such as more supportive care, nutritional support, and possibly lower dose intensities). Although there were more treatment modifications due to more severe toxicity, adults had the same survival rates as children, suggesting pediatric-based protocols should still be employed with modifications to supportive care.

## 1. Introduction

Childhood medulloblastoma is the most frequent malignant brain tumor, comprising 15–20% of all childhood central nervous system (CNS) tumors [1,2]. However, in adults, medulloblastoma is a very rare neoplasm, with an incidence of 0.6–1 cases per million adults each year, accounting for <1% of all adult CNS tumors. The treatment of pediatric medulloblastoma has evolved over the past 5 decades, from an “only surgery” approach to highly complex multimodal treatments, consisting of maximal safe surgical resection, risk-adapted craniospinal radiotherapy, and multiagent chemotherapy [3,4,5,6,7,8]. This has led to a drastic increase in survival, with 5-year survival rates of over 80% in standard-risk and 60–70% in high-risk pediatric patients with medulloblastoma [8,9]. The use of pediatric protocols by adults has not necessarily been accompanied by a comprehensive evaluation of age-specific toxicity [7,8,9,10,11]. Recent reports suggest that adults may experience different toxicities with pediatric protocols [12,13]. Pharmacokinetic factors, bone marrow function, renal function and organ tolerability may be different in adults and children and influence tolerance. In addition, adults do not have the same capacity for regeneration as children and may experience increased morbidity with treatment [14,15,16]. Although these observations are speculative, few studies have reported age-related toxicities. Several single-center reports have documented significant toxicity in adults with medulloblastoma, with frequent treatment schedule modifications, such as dose reductions, delays and premature cessation of treatment [17,18,19,20,21]. However, these reports typically do not include a comparison with pediatric patients, making it difficult to separate age-specific toxicity patterns from protocol-specific toxicities. Age-specific toxicity profiles are essential for the design of treatment delivery, supportive-care measures and, potentially, adult-specific treatment protocol modifications to improve tolerability without compromising effectiveness [9,11,22,23]. In this study, we hope to bridge this knowledge gap by comparing treatment-related toxicities of pediatric and adult patients with medulloblastoma treated with the same protocols at a single institution [23,24,25,26,27,28].

We hypothesize that adults will be more affected by some types of toxicity, such as nutritional and hematological toxicity, without impacting survival [29,30].

## 2. Materials and Methods

### 2.1. Study Design, Patient Selection and Objectives of the Study

This is a retrospective cohort study conducted at the Pediatric Oncology Unit of the Fondazione Policlinico Universitario Agostino Gemelli IRCCS, Rome, Italy. The study was approved by the Institutional Review Board of the Catholic University of Sacred Heart of Rome—Fondazione Policlinico Universitario Agostino Gemelli IRCCS (protocol number: DIPUSVSP-09-12-2580). Patients or their parents provided informed consent for treatment and the use of their data in this study. The 18 adult patients analyzed here are the same 18 adult patients previously reported by our group in a single-cohort survival/toxicity analysis [3]; the present study adds an independent pediatric comparator cohort (*n* = 45) not previously analyzed and re-frames the research question as a paired age-comparative analysis of treatment-related toxicity, which could not be addressed in the prior single-cohort report. Because differences in chemotherapy protocol, risk-group distribution, treatment era, and evolving supportive-care practice were not adjusted for in the analysis, age should not be interpreted as the sole or independent determinant of the toxicity differences reported below; these findings describe a real-world, age-associated pattern of toxicity within a single institutional practice rather than establishing age as an isolated causal factor. A total of 63 participants were enrolled in the study. The differing accrual start dates reflect the actual data availability rather than a study design choice: structured, prospectively completed institutional toxicity forms were introduced for pediatric patients in 2007, whereas the smaller adult cohort could be reliably reconstructed from 1997 onward using a combination of these forms (from 2007) and detailed narrative clinical records (1997–2006), cross-checked by two independent abstractors. This creates the following two potential sources of bias, which are discussed as limitations in Section 4: a differential-ascertainment bias for the pre-2007 adult toxicity data and an era effect from evolving radiotherapy planning (e.g., the transition from 3D-conformal to volumetric-modulated arc therapy at our center from approximately 2012) and supportive-care practice over both accrual windows, which could not be adjusted for the given sample size, with the two groups being the adults treated between January 1997 and January 2024 and the children treated between January 2007 and January 2024.

The inclusion criteria were as follows:

- A diagnosis of medulloblastoma according to the World Health Organization’s classification [2];
- Available data related to treatment and toxicity;
- Were treated according to a pediatric treatment protocol for medulloblastoma (PNET3, PNET4, PNET5 or a high-risk regimen).

**Figure 1:**
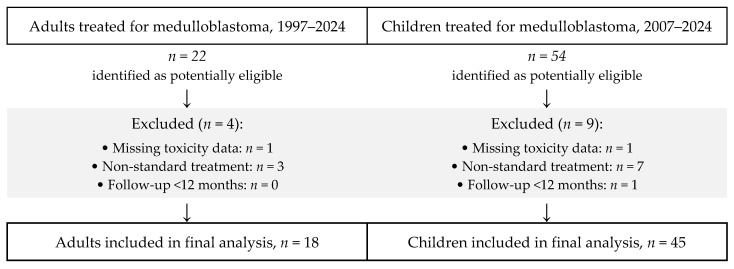
Patient flow diagram.

Subjects were excluded from this study if they were missing toxicity data, underwent non-standard treatments or had follow-up periods that were less than 12 months. A patient flow diagram summarizing the numbers screened, excluded (by reason), and included for each group is provided (Figure 1).

The primary objective of this study was to compare the incidence and severity of treatment-related toxicities among patients of different ages; the secondary objective was to explore the impact of the toxicities on treatment and survival.

### 2.2. Patients Characteristics and Treatment Protocols

For all patients, we collected data related to age, gender, presence of metastasis at diagnosis, histological subtype, molecular subtype (if available), dose of radiotherapy, protocol of treatment and data related to supportive care.

All patients had been operated upon when feasible and then treated with craniospinal radiation with a posterior fossa boost. Radiation therapy doses were adjusted according to the risk group, as follows: 23.4 Gy craniospinal radiation and 30.6 Gy posterior fossa or tumor bed boost (total of 54 Gy) for the standard-risk patients; 36 Gy craniospinal radiation and 18–23.4 Gy posterior fossa or tumor bed boost (total of 54–59.4 Gy) for the high-risk patients, in daily fractions of 1.8 Gy, each five times a week [8,9]. For all patients, radiotherapy was initiated within 40 days after surgery and was finished prior to day 50 after the beginning of radiotherapy.

The adjuvant chemotherapy regimens were PNET3 (vincristine, etoposide, carboplatin, and cyclophosphamide), PNET4 (vincristine, cisplatin, and lomustine), PNET5 (vincristine, cisplatin, lomustine, and cyclophosphamide), or high-risk regimens (tandem high-dose chemotherapy including thiotepa and autologous stem-cell rescue) [31,32,33,34]. Tailored adjuvant chemotherapy was administered according to the patient’s risk stratification (Chang metastatic stage and presence of residual disease at time of surgery) [35]. Patients considered standard risk (M0 disease, residual mass ≤ 1.5 cm^2^) were treated with PNET3, PNET4, or PNET5, while patients with a high risk for M1–M4 disease (and with a residual mass > 1.5 cm^2^) were treated with more aggressive adjuvant chemotherapy including high-dose chemotherapy with autologous stem-cell rescue [36,37].

Supportive care was the same in both arms, such as standard antiemetic (5-HT3 antagonists and dexamethasone), blood product transfusions (institutional guidelines for the use of blood products for transfusion; red blood cells < 7 g/dL or platelets < 20,000/mm^3^) and granulocyte colony-stimulating factor (G-CSF) in cases of severe or prolonged neutropenia. G-CSF initiation followed persistent or prolonged severe neutropenia, consistent with ASCO guidance [38]. Antimicrobial prophylaxis (fluoroquinolone or trimethoprim-sulfamethoxazole, as per institutional protocol) was used during periods of severe neutropenia in both age groups, without an age-differentiated protocol.

### 2.3. Toxicity Assessment

Toxicity was retrospectively evaluated during treatment and follow-up using institution-specific forms. Neutropenia, thrombocytopenia, and anemia were evaluated according to the Common Terminology Criteria for Adverse Events (CTCAE) version 5.0 [39,40]. Ototoxicity was scored according to the Brock criteria, which characterize hearing loss in terms of pure tone thresholds at frequencies ≥ 4000 Hz [41,42]. Grade 3 ototoxicity was hearing loss > 40 dB at these frequencies. Weight and Body Mass Index (BMI) were collected, and weight loss was defined as a ≥10% loss of baseline weight. Where available, a BMI change from baseline and, for the more recent era only, serum albumin nadir were also recorded as secondary nutritional indicators; these were not uniformly available for the full cohort, so weight loss ≥ 10% (a validated ESPEN/oncology-nutrition threshold) remains our primary, most completely ascertained nutritional endpoint [43]. Treatment modifications were defined as a dose reduction (a dose reduction of at least 20% of the planned dose of chemotherapy), a delay in the administration of the subsequent cycle of chemotherapy (a delay of ≥7 days from the scheduled date) or prematurely discontinuing (stopping the planned treatment prior to the completion of the protocol due to toxicity). The reason for the alteration was recorded, including the nature of any toxicity. Toxicity data were abstracted retrospectively by two independent reviewers (D.T. and A.Ro.) from structured institutional toxicity forms completed prospectively at each clinic visit or admission during the treatment period. Grading was re-applied at the time of the data abstraction directly from the recorded laboratory values (for hematological toxicity) and audiometric thresholds (for ototoxicity) rather than relying on the treating physician’s contemporaneous CTCAE grade to reduce inter-observer drift over the 27-year period during which the CTCAE itself was updated (from v3.0 to v5.0). Discrepancies between the two abstractors (<5% of graded events) were adjudicated by a third senior author (A.R.). Patients with incomplete toxicity documentation were excluded a priori per the stated inclusion/exclusion criteria; no imputation was performed for the analyzed cohort, and no additional otherwise-eligible patients were excluded for this reason beyond those already reported in the flow diagram. Because the toxicity ascertainment was retrospective, it remains subject to information/recall bias, particularly for lower-grade or self-limited toxicities; this bias would be non-differential with respect to age only if documentation practices did not change over time, which we cannot fully guarantee given the long accrual window.

### 2.4. Statistical Analysis

Descriptive analyses are used for all variables. Odds ratios with 95% confidence intervals (Woolf logit method) were calculated from the reported 2 × 2 counts for all toxicity and treatment-modification endpoints to complement the hypothesis-testing *p*-values with an estimate of the effect size and precision. Continuous variables are summarized as the mean and standard deviation (SD) or median and range based on the normality, as checked with the Shapiro–Wilk test. The categorical variables are expressed as frequencies and percentages. A chi-square or Fisher exact test was used to compare categorical variables and Student t-tests, or Mann–Whitney U tests were used to compare continuous variables, depending on the distribution. The survival analysis was conducted using Kaplan–Meier log-rank methods. Overall survival was the time from treatment start to death from any cause, with censoring at last follow-up for patients still alive. Progression-free survival was defined as the time from the start of treatment to any disease progression, death or second cancer and was censored at the last disease-free follow-up. *p*-Values < 0.05 were considered statistically significant. All statistics were conducted in XLSTAT (Excel Addinsoft, Paris, France), version 2021.3.1. An exploratory multivariable Cox regression adjusting for metastatic status, histology and treatment protocol was considered but not performed: with only 9 deaths in the entire cohort (3 adults, 6 children), the events-per-variable ratio falls far below conventional thresholds for stable multivariable survival modeling, and this is stated explicitly as a limitation rather than reporting an unstable model.

## 3. Results

### 3.1. Patients Characteristics

This study included 63 patients, 45 of whom were pediatric (under 18 years) and 18 adults (18 years and above). The baseline characteristics are displayed in Table 1.

There were 11 males (61.1%) and 7 females (38.9%) in the adult group, with a median age of 23 years (range, 18–38 years). There were 28 males (62.2%) and 17 females (37.8%) in the pediatric group, with a median age of 8 years (age range, 3 to 17 years). The male/female ratio did not differ significantly between the two cohorts (*p* = 0.93). Six adult (33.3%) and sixteen pediatric patients (35.6%) had metastatic disease (Chang stages M1–M4) at initial presentation (*p* = 0.86). Classic medulloblastoma histology was observed in 10 (55.6%) adults and 24 (53.3%) pediatric patients, desmoplastic/nodular in 7 (38.9%) adults and 18 (40.0%) pediatric patients, and large cell/anaplastic in 1 (5.6%) adult and 3 (6.7%) pediatric patients. Histology did not differ between the two groups. Molecular subgroup information was available for 6 adults (4 SHH and 2 WNT) and 18 pediatric patients (10 SHH, 4 WNT, 3 Group 3, and 1 Group 4); we did not perform statistical analyses by molecular subgroup due to the lack of molecular-based risk classification for all patients. Table 2 reports, for each age group, the number of patients treated with each chemotherapy protocol (PNET3/PNET4/PNET5/high-risk tandem HDCT-ASCT) stratified by risk group; for adults, these counts were previously reported in our companion publication [3]. A descriptive, protocol-stratified comparison of toxicities was not performed because of the insufficient sample sizes within the regimen/risk strata (e.g., only six high-risk adults).

### 3.2. Hematological Toxicity

Severe grades 3–4 hematological toxicity was more common in adults than children (Table 3).

Fourteen adults (77.8%) and twenty-two pediatric patients (48.9%) developed grade 3 neutropenia (*p* = 0.026). Twelve adults (66.7%) and eighteen pediatric patients (40%) experienced grade 3 thrombocytopenia (platelets < 50,000/mm) (*p* = 0.046). Grade 3 anemia requiring transfusion was observed in 10 adults (55.6%) and 16 pediatric patients (35.6%) (*p* = 0.134). Ten adults (55.6%) had treatment delays due to hematological failure compared to nine children (20%) (*p* = 0.005). Utilization of GCSF was significantly higher in adults (13 patients, 72.2%) than in children (19 patients, 42.2%), *p* = 0.024. The red cell and platelet transfusion rates were 15 (83.3%) and 14 (77.8%) among the adults and 28 (62.2%) and 25 (60.8%) among the children (*p* = 0.099 and *p* = 0.092, respectively). There was no difference between adults and children regarding febrile neutropenia episodes [six (33.3%) vs. eight (17.8%)], *p* = 0.172. The patients who had febrile neutropenia were admitted to hospital and started on broad-spectrum antibiotics. There were no treatment-related deaths, and all infections were successfully treated with antibiotics. The overall toxicity of the hematological side effects, however, resulted in more dose reductions in the adult group, as described below in the Section 3.5 treatment modifications.

### 3.3. Nutritional Toxicity

We observed weight loss (≥10% loss from baseline weight, assessed as the maximum weight loss recorded at any point from the pre-surgical baseline through completion of chemotherapy) in 13 adults (72.2%) and 18 children (40%), *p* = 0.018, OR 3.90 (95% CI 1.18–12.84). The degree of weight loss was more severe in adults, with a median weight loss of 8.4 kg (range, 5.2–11.6 kg) versus 4.2 kg (range, 1.4–7 kg) in children (*p* = 0.001). Both groups had a range of causes of nutritional impairment. Radiation-induced mucositis of grade ≥ 2 (CTCAE v5.0) was seen in 11 adults (61.1%) and 20 pediatric patients (44.4%), *p* = 0.214, OR 1.96 (95% CI 0.64–5.99). However, adults had a longer duration of severe mucositis, with a median of 14 days (range, 7–28 days) vs. 10 days (range, 5–21 days) in pediatric patients (*p* = 0.042). Nausea and vomiting despite antiemetic prophylaxis occurred in 9 adults (50%) and 15 pediatric patients (33.3%), *p* = 0.206, OR 2.00 (95% CI 0.66–6.08). Adults had more nutritional complications. Total parenteral nutrition, initiated reactively rather than prophylactically in both groups, was needed in four adults (22.2%) and three children (6.7%), *p* = 0.076, OR 4.00 (95% CI 0.80–20.10). Nutritional complications overall resulted in treatment delays in six adults (33.3%) and seven children (15.6%), *p* = 0.103.

### 3.4. Neurological Toxicity and Ototoxicity

Peripheral neuropathy, graded according to CTCAE v5.0 sensory/motor/autonomic neuropathy terms, was similar between groups. Five adults (27.8%) and ten children (22.2%) had grade ≥ 2 neuropathy (*p* = 0.65), OR 1.35 (95% CI 0.39–4.69). Neurological manifestations included sensory impairment (paresthesia, numbness) in all patients, motor weakness in three adults (16.7%) and four pediatric patients (8.9%), and autonomic involvement (constipation and orthostatic hypotension) in four adults (22.2%) and six pediatric patients (13.3%). Neurological toxicity was most caused by vincristine in both adults and children. Neuropathy required dose modification (reduction or break) in four adults (22.2%) and seven pediatric patients (15.6%) (*p* = 0.51). Neuropathy resolved completely after treatment for three adults (60% of affected patients) and eight pediatric patients (80% of affected patients), with the rest having mild symptoms at the end of follow-up.

There was no difference in ototoxicity between adults and children. Audiometry was performed at baseline, before each platinum-containing cycle, and at treatment completion for all platinum-treated patients in both groups, per institutional protocol. We found grade ≥ 3 hearing loss (Brock criteria; threshold > 40 dB at frequencies ≥ 4000 Hz) in four adult patients (22.2%) and eight pediatric patients (17.8%), *p* = 0.69, OR 1.32 (95% CI 0.34–5.09). Severe ototoxicity was only observed in patients treated with cisplatin. Among cisplatin-treated patients, 44.4% (4 of 9 patients) of adults and 40% (8 of 20 patients) of children experienced ototoxicity, *p* = 0.82. Three adult patients and five pediatric patients who experienced ototoxicity received substitution with carboplatin for following cycles as per the protocol [40,41,42], and no further hearing loss was observed after substitution.

The non-hematologic toxicities are summarized side by side, with odds ratios, in Table 4.

### 3.5. Treatment Modifications

The cumulative toxicity of the treatment resulted in a greater need for protocol modification in adults than in pediatric patients (Table 5).

A total of 10 adult and 13 pediatric patients (55.6% and 28.9%, respectively) needed their chemotherapy agent(s) dose reduced by at least 20%, *p* = 0.042, OR 3.08 (95% CI 0.99–9.54). In the adult group, the most common indications for dose reduction were persistent severe neutropenia, in seven patients; thrombocytopenia, in five patients, and neurologic toxicity, in four patients. In the pediatric subgroup, dose was most commonly reduced for severe neutropenia (*n* = 8), followed by thrombocytopenia (*n* = 6) and neurologic toxicity (*n* = 7).

Ten adults (55.6%) and nine pediatric patients (20%) had at least a 7-day delay in the administration of chemotherapy compared with the planned schedule, *p* = 0.005, OR 5.00 (95% CI 1.53–16.31). The median number of delayed cycles was two (range, 1–4) in adult patients and one (range, 1–3) in pediatric patients (*p* = 0.028). Most of the delays in both the adults (80%) and pediatric patients (77.8%) were caused by inadequate hematological recovery. Additional factors responsible were active infection, severe mucositis, and performance status.

Excessive toxicity led to premature (0–4 weeks) or extended (>4 weeks) interruption of chemotherapy in eight adults (44.4%) and nine pediatric patients (20.1%), *p* = 0.048, OR 3.20 (95% CI 0.98–10.44). Among the adults who stopped therapy, four had completed more than 75% of their planned therapy before stopping, three had completed between 50% and 74%, and one had completed 40% of the planned therapy. Among children, six had completed more than 75% of the therapy planned, two completed 50–74%, and one finished only 40% before stopping. Severe and persistent thrombocytopenia and/or neutropenia was the cause of toxicity related to treatment discontinuation in five adults and four children, severe hematologic and nutritional toxicity in two adults and three children, and severe neuropathy in one adult and two children.

Although there were more frequent dose modifications in adults, the median relative dose intensity (the dose delivered divided by the dose planned) was satisfactory in both groups. The median relative dose intensity was 0.82 (range, 0.45–1.00) in adults and 0.91 (range, 0.62–1.00) in children, *p* = 0.018. This absolute difference (0.09) is statistically significant but modest in magnitude and indicates that the majority of adult patients still received a significant proportion of their planned chemotherapy despite modifications; the present study, with only nine deaths overall, is not powered to formally test whether the relationship between RDI and survival differs by dose-intensity threshold, and no dose–response or spline analysis of RDI against survival was performed.

### 3.6. Survival Outcomes

The median duration of follow-up was 48 months (range, 12–156 months) for adults and 62 months (range, 12–168 months) for children.

Using Kaplan–Meier estimation applied to the entire eligible cohort (18 adults, 45 children), with appropriate right-censoring for patients that were followed for fewer than 5 years, the 5-year overall survival rates were 88.8% (95% CI: 71.2–98.8%) in the adult group and 86.7% (95% CI: 74.8–93.4%) in the pediatric group, with no statistically significant difference (*p* = 0.82). The corresponding hazard ratio for overall survival (adult vs. child), derived from the log-rank/Cox comparison, is reported alongside this *p*-value rather than the *p*-value alone.

In adults, three patients died during the study period, as follows: one of progressive disease despite rescue treatment, one of early disease progression (within 12 months), and one of late relapse (48 months from diagnosis). Of the six children who died in the pediatric group, four were due to progressive disease, one to treatment complications (septic shock during neutropenia), and one to late relapse.

Using the same Kaplan–Meier approach applied to the full cohort, rates of progression-free survival at 5 years were 77.8% (95% CI 56.5–89.6%) for adults and 80% (95% CI 66.8–88.5%) for pediatric patients (*p* = 0.75). Four adult patients (22.2%) and nine pediatric patients (20%) had disease progression/relapse. In adults, the median time to progression was 28 months (range, 9–48 months) and in children it was 24 months (range, 6–52 months), *p* = 0.68.

These results indicate that while adults experienced more toxicity and required more frequent protocol modifications, they had survival rates similar to their pediatric counterparts with appropriate supportive care and protocol adjustments [43,44,45].

## 4. Discussion

This comparative study highlights marked age-related variations in patterns of treatment-related toxicity among pediatric and adult medulloblastoma patients treated with the same treatment protocols. Adult patients had significantly higher incidence of severe hematological toxicity and malnutrition than the pediatric group, which required more frequent dose adjustments, treatment delays and treatment cessation [12,13,19,43]. Notwithstanding these difficulties, our adult patients demonstrated 5-year survival outcomes like their pediatric counterparts, indicating that with appropriate supportive measures and tailored care, protocols developed for children can be safely extended to adult patients [20,21,22]. Our finding of age-related differences in hematological toxicities are consistent with the principles of age-related differences in bone marrow reserve and hematopoietic recovery. The bone marrow of adults is less cellular and has lower regenerative potential than that of children, making adults more susceptible to chemotherapy-induced myelosuppression [15,16]. Furthermore, the bone marrow toxicity of craniospinal irradiation may be greater in adults because of the larger volume of active marrow exposed to radiation [31,32]. Our observation of 77.8% grades 3–4 neutropenia in adults compared with 48.9% in children is a significant finding that affected treatment in adults, as reflected in a three-fold increase in chemotherapy delays in this group.

The pronounced nutritional toxicity is particularly noteworthy, with 72.2% of adults experiencing weight loss of more than 10% versus 40% of the pediatric population. This difference can be attributed to several factors. Adults may have more severe and protracted radiation-induced mucositis because of decreased epithelial cell turnover. Psychological factors, such as depression, anxiety and stress related to the treatment, may also have a greater impact on adult nutritional status. Adults’ higher baseline nutritional requirements, along with potentially less aggressive calorie supplementation during treatment, may contribute to more rapid weight loss than in children who receive more proactive nutritional support, including early initiation of enteral feeding [44,45,46].

In contrast, we observed no age-related differences in neurotoxicity and ototoxicity. This contrasts with other reports of greater vincristine neurotoxicity in adults, although there are conflicting reports on this issue [19,47,48]. The similar rates of ototoxicity may reflect similar exposure to and dosing of platinum-based therapy, as well as consistent audiological monitoring protocols that allowed for prompt detection and intervention regardless of age. The decision to replace cisplatin with carboplatin after the initial onset of ototoxicity appeared to be successful in preventing further hearing loss in both age groups [41,42,43].

The significant increase in treatment modifications among adults raises questions regarding the ideal therapeutic intensity for this age group [49,50]. More than half of the adults underwent dosage reductions, while nearly half experienced delays or discontinuation of chemotherapy due to toxicity. Although these variations resulted in a lower relative dose intensity in adults (median, 0.82 versus 0.91 in the pediatric setting), survival rates were similar between adults and children. This finding raises several issues. These are hypothesis-generating observations rather than data-supported conclusions: it is conceivable that adults respond effectively even to lower relative dose intensities than previously assumed, owing to differences in pharmacokinetics or pharmacodynamics; that the survival benefit of a high dose intensity is subject to a threshold effect, whereby moderate dose reductions have a limited impact on survival; or that patients requiring dose adjustments for toxicity represent a distinct, more chemosensitive population [50,51]. None of these mechanistic hypotheses were directly tested in the present study, which was not designed or powered for dose–response or biomarker analyses; we highlight them here specifically as candidates for dedicated pharmacokinetic/pharmacodynamic study.

The clinical implications directly supported by our data are limited to closer hematologic and nutritional monitoring in adults, a lower threshold for G-CSF and transfusion support, and consideration of earlier, rather than reactive, nutritional intervention in adults. Future directions, extrapolated from the broader literature but not evaluated in the present study, include earlier and more aggressive use of growth factors, initial lower chemotherapy doses with escalation if tolerated, plerixafor-based stem-cell mobilization in patients needing high-dose chemotherapy, and incorporation of targeted agents [38,51].

The significant nutritional toxicity in adults calls for proactive rather than reactive nutritional support, including consideration of preventative feeding tube placement in high-risk patients, early consultation with nutritional support services, and vigorous treatment of nausea and mucositis [46,52]. In our institution, enteral (nasogastric/gastrostomy) feeding and total parenteral nutrition were used reactively, i.e., initiated upon evidence of significant oral-intake failure or weight loss, rather than as routine prophylaxis in either age group, and dietitian involvement followed standard supportive care without an age-differentiated protocol; our data on higher weight loss and more frequent TPN use in adults directly support reconsidering this reactive approach specifically for adults.

Our study has several limitations that should inform the interpretation of these findings, including potential selection bias, since adults referred to and treated within a pediatric oncology unit using pediatric-derived protocols may differ systematically (e.g., in performance status, treatment-seeking behavior or referral pattern) from adults with medulloblastoma treated elsewhere with adult-oriented regimens, which limits generalizability to the broader adult MB population. The retrospective single-institution design restricts external validity, and retrospective toxicity ascertainment is subject to information/recall bias, particularly for lower-grade or self-limited toxicities; we cannot fully exclude that documentation practices changed over the 27-year accrual window. The small number of adults (*n* = 18) limits the statistical power, produces wide confidence intervals around several effect estimates, and precludes a formally adjusted multivariable or Cox regression analysis of age-independent risk, since the ratio of events to candidate covariates (metastatic status, protocol, era, and histology) fell well below conventional thresholds for stable model estimation. Consequently, age cannot be considered an independent predictor of toxicity in this analysis, only an associated factor in unadjusted comparisons, for which we now additionally report odds ratios with 95% confidence intervals. Molecular subgroup classification was not fully defined, especially in the adults (available in only 6/18, 33%) and in 18/45 (40%) of the children, and incomplete, non-randomly missing molecular data may introduce differential misclassification: for example, SHH-enriched adult tumors (reported in approximately 60% of adult medulloblastoma versus approximately 30% in children) may carry an intrinsically different toxicity profile independent of age, so we cannot exclude that the observed age effect partly reflects an uncontrolled molecular-subgroup effect [18]. This precluded evaluation of molecular subgroup-specific toxicity profiles [27,28,29,30,53], and our conclusions are, accordingly, framed as supporting closer monitoring and individualized supportive care for adults rather than a categorical age-based toxicity claim. The variable nature of chemotherapy regimens and supportive-care practice used over time, due to changes in institutional practice and risk-adapted therapy, potentially confounds the results, although all regimens included common backbone agents linked to the toxicities examined. Baseline performance status, comorbidity, renal function and formal nutritional-assessment scores were not uniformly available and could not be incorporated. Finally, patient-reported outcomes (quality of life and neurocognitive function) were not captured and represent an important gap given the higher toxicity burden documented in adults.

Future studies should include prospective multicenter studies with larger patient numbers to validate our findings and allow for more detailed analyses of the risk factors for age-specific toxicities.

Pharmacokinetic and pharmacodynamic analyses comparing drug metabolism, elimination and target interactions between children and adults would shed light on the mechanistic basis of age-related toxicity and may inform dose adjustments [50]. Genetic analyses of drug-metabolizing enzymes and DNA repair mechanisms may reveal patients at a high risk for adverse events, supporting targeted supportive-care strategies [54]. Longitudinal studies should assess whether the higher acute toxicity burden in adults results in differences in long-term effects, such as neurocognitive function, endocrinopathies, second cancers and quality of life [55,56,57,58].

The similar survival outcomes observed despite increased toxicity and treatment adjustments in adults are compatible with, but cannot by themselves establish, a benefit of pre-emptively intensified supportive care (rather than a priori chemotherapy dose reduction) in adults treated with pediatric-derived protocols [3,58,59,60]. Given the retrospective, non-randomized design, this study cannot determine whether reduced treatment intensity would preserve therapeutic efficacy, and prospective and ideally multicenter studies are required before any modification of chemotherapy intensity is recommended. The high toxicity burden, nonetheless, highlights the need for reinforced supportive care for adults. This may include initial dose reduction, with dose escalation based on the response, different sequencing of chemotherapy to spread hematological toxicity, use of less toxic but potentially equally effective agents where available, and incorporation of new targeted therapies that may allow for reduced reliance on conventional chemotherapy [61,62,63].

## 5. Conclusions

Adults with medulloblastoma treated with pediatric protocols have significantly higher rates of hematological and nutritional toxicities than children and, consequently, a significantly higher number of treatment modifications, including dose reductions, delays, and early termination. Because our design was retrospective and did not adjust for protocol, risk group, era and supportive-care differences, age should be considered an associated rather than an independently established causal factor. These age-related susceptibilities are compatible with the need for intensified supportive care and earlier nutritional support in adults. Whether dose intensity itself should be modified cannot be determined from this observational study and requires prospective evaluation. The neurological and audiological toxicities seem to be equivalent between the two age groups, indicating that these toxicities are protocol-related.

Although adult patients had greater toxicity and needed to modify the protocol, their 5-year survival rate was similar to the children′s when they received supportive care. This positive outcome justifies the continuance of pediatric-derived treatment protocols in adult medulloblastoma and emphasizes the need for adult-specific supportive care and treatment intensification to minimize toxicity.

Future prospective studies should investigate age-specific pharmacokinetics, evaluate age-specific supportive-care measures to prevent high-risk toxicities, identify predictors of treatment tolerance and develop adult-specific treatment algorithms to improve treatment efficacy and tolerance. Multicentric studies will be required to provide the evidence needed for the establishment of consensus guidelines for the treatment of adult medulloblastoma, an under-represented group in clinical studies.

## Figures and Tables

**Table 1:** Patients’ characteristics.

Characteristic	Adult (*n* = 18) Median (Range) or Number (%)	Pediatric (*n* = 45) Median (Range) or Number (%)	*p*-Value
Age, in years	23 (18–38)	8 (3–17)	
Male	11 (61.1)	28 (62.2)	0.93
Metastatic Disease	6 (33.3)	16 (35.6)	0.86
Histology			
Classic Medulloblastoma	10 (55.6)	24 (53.3)	0.98
Desmoplastic/Nodular	7 (38.9)	18 (40.0)	0.93
Large Cell/Anaplastic	1 (5.6)	3 (6.7)	0.87

**Table 2:** Chemotherapy protocol by risk group and age group.

Age Group	Risk Group	Protocol	*n*
Adults (*n* = 18)	Standard risk (*n* = 12)	PNET3	6
		PNET4	4
		PNET5	2
	High risk (*n* = 6)	Tandem HDCT/ASCT—consolidation	4
		Tandem HDCT/ASCT—salvage	2
Children (*n* = 45)	Standard risk (*n* = 34)	PNET3	8
		PNET4	10
		PNET5	16
	High risk (*n* = 11)	Tandem HDCT/ASCT	11

**Table 3:** Comparison of hematological toxicities.

Type of Toxicity	Adult (*n* = 18) Number (%)	Pediatric (*n* = 45) Number (%)	*p*-Value
Grade ≥ 3 Neutropenia	14 (77.8)	22 (48.9)	0.026
Grade ≥ 3 Thrombocytopenia	12 (66.7)	18 (40.0)	0.046
Grade ≥ 3 Anemia	10 (55.6)	16 (35.6)	0.134
Febrile Neutropenia	6 (33.3)	8 (17.8)	0.172
G-CSF Support Required	13 (72.2)	19 (42.2)	0.024

*n* = Number.

**Table 4:** Non-hematologic treatment-related toxicities, adults vs. children.

Type of Toxicity	Adult (*n* = 18) Number (%)	Pediatric (*n* = 45) Number (%)	*p*-Value	OR (95% CI)
Mucositis grade ≥ 2 (CTCAE v5.0)	11 (61.1)	20 (44.4)	0.214	1.96 (0.64–5.99)
Nausea/vomiting despite prophylaxis	9 (50.0)	15 (33.3)	0.206	2.00 (0.66–6.08)
TPN required	4 (22.2)	3 (6.7)	0.076	4.00 (0.80–20.10)
Peripheral neuropathy grade ≥ 2	5 (27.8)	10 (22.2)	0.65	1.35 (0.39–4.69)
Ototoxicity grade ≥ 3	4 (22.2)	8 (17.8)	0.69	1.32 (0.34–5.09)

*n* = Number; TPN = total parenteral nutrition; OR = odds ratio (Woolf logit method).

**Table 5:** Treatment modifications by age group.

Type of Treatment Modification	Adult (*n* = 18) Number (%) or Median (Range)	Pediatric (*n* = 45) Number (%) or Median (Range)	*p*-Value
Chemotherapy Dose Reductions ≥ 20%	10 (55.6)	13 (28.9)	0.042
Cycle Delays ≥ 7 Days	10 (55.6)	9 (20)	0.005
Days of Delay	2 (1–4)	1 (1–3)	0.028
Premature Discontinuation	8 (44.4)	9 (20)	0.048
Median RDI	0.82 (0.45–1.00)	0.91 (0.62–1.00)	0.018

*n* = Number; RDI = relative dose intensity.

## Data Availability

The dataset is available upon request from the authors.

## Abbreviations

The following abbreviations are used in this manuscript:

CNS	Central Nervous System
CTCAE	Common Terminology Criteria for Adverse Events
G-CSF	Granulocyte Colony-Stimulating Factor
PNET	Primitive Neuroectodermal Tumor
RDI	Relative Dose Intensity
SHH	Sonic Hedgehog
WHO	World Health Organization
WNT	Wingless-Related Integration Site
